# Capillary morphogenesis gene 2 maintains gastric cancer stem-like cell phenotype by activating a Wnt/β-catenin pathway

**DOI:** 10.1038/s41388-018-0226-z

**Published:** 2018-04-17

**Authors:** Chengdong Ji, Lang Yang, Weijing Yi, Dongfang Xiang, Yanxia Wang, Zhihua Zhou, Feng Qian, Yong Ren, Wei Cui, Xia Zhang, Peng Zhang, Ji Ming Wang, Youhong Cui, Xiuwu Bian

**Affiliations:** 1 Institute of Pathology and Southwest Cancer Center, and Key Laboratory of Tumor Immunopathology of Ministry of Education of China, Southwest Hospital, Third Military Medical University (Army Medical University), 400038 Chongqing, China; 2 Department of Pathology, The 101 Hospital of People’s Liberation Army, Wuxi, 214000 Jiangsu Province China; 3 Department of General Surgery, Southwest Hospital, Third Military Medical University, 400038 Chongqing, China; 4 Cancer and Inflammation Program, Center for Cancer Research, National Cancer Institute at Frederick, Frederick, MD 21702 USA

## Abstract

A growing body of evidence shows that the development and progression of gastric cancer (GC) is mainly associated to the presence of gastric cancer stem-like cells (GCSLCs). However, it is unclear how GCSLC population is maintained. This study aimed to explore the role of capillary morphogenesis gene 2 (CMG2) in GCSLC maintenance and the relevance to GC progression. We found that CMG2 was highly expressed in GC tissues and the expression levels were associated with the invasion depth and lymph node metastasis of GC, and inversely correlated with the survival of GC patients. Sorted CMG2^High^ GC cells preferentially clustered in CD44^High^ stem-like cell population, which expressed high levels of stemness-related genes with increased capabilities of self-renewal and tumorigenicity. Depletion of *CMG2* gene resulted in reduction of GCSLC population with attenuated stemness and decrease of invasive and metastatic capabilities with subdued epithelial–mesenchymal transition phenotype in GC cells. Mechanistically, CMG2 interacted with LRP6 in GCSLCs to activate a Wnt/β-catenin pathway. Thus, our results demonstrate that CMG2 promotes GC progression by maintaining GCSLCs and can serve as a new prognostic indicator and a target for human GC therapy.

## Introduction

Gastric cancer (GC) is the third leading cause of cancer-related death worldwide [1, 2]. The 5-year overall survival rate of GC patients remains lower than 40%, mainly due to cancer invasiveness and metastasis [3, 4]. Recent studies suggested that gastric cancer stem-like cells (GCSLCs) are responsible for the invasion and metastasis [5–7], and thus targeting GCSLCs has become a promising therapeutic strategy for GC. However, the molecular mechanisms underlying GCSC maintenance is largely unknown.

CMG2 is a single transmembrane protein induced during capillary morphogenesis [8]. CMG2 is also known as anthrax toxin receptor 2 (ANTXR2) because it functions as a receptor for anthrax toxin similar to its paralog ANTXR1 (TEM8) [9, 10]. Until now, the physiological function of CMG2 is poorly understood. It has been reported that CMG2 accumulates in the cortical actin cap along the embryonic A-V axis by interacting with actin to orient cell mitosis during the embryogenesis of zebrafish [11]. Based on the presence of an extracellular von Willebrand A (vWA) domain, CMG2 is proposed to bind collagen IV and laminin, suggesting a potential role in basement membrane matrix synthesis and assembly [8]. Recently, CMG2 was demonstrated to act as a receptor for collagen VI and mediate its intracellular degradation [12]. Mutations in CMG2 result in the allelic disorders juvenile hyaline fibromatosis and infantile systemic hyalinosis characterized by multiple, recurring subcutaneous tumors, gingival hypertrophy, joint contractures, osteolysis, and osteoporosis [13]. In tumors, CMG2 is involved in the angiogenic processes by promoting endothelial proliferation and morphogenesis [14–16]. CMG2 plays contradictory roles in cells of prostate cancer [17], breast cancer [18], and glioma [19]. In our expression, chip analysis of GC tumor-sphere cells, which possessed the characteristics of GCSLCs [20], CMG2 was found to be markedly overexpressed in GC tumor-sphere-forming cells, suggesting that CMG2 may play an important role in GCSLC maintenance.

We therefore investigated the role of CMG2 in regulating GCSLC properties and its clinical relevance to human GC. We found that CMG2 maintains GCSLC population and can act as an independent indicator of GC prognosis as well as a potential target for GC therapy.

## Results

### CMG2 is highly expressed in GC tissues and the expression is correlated with the outcome of patients

**Fig. 1:**
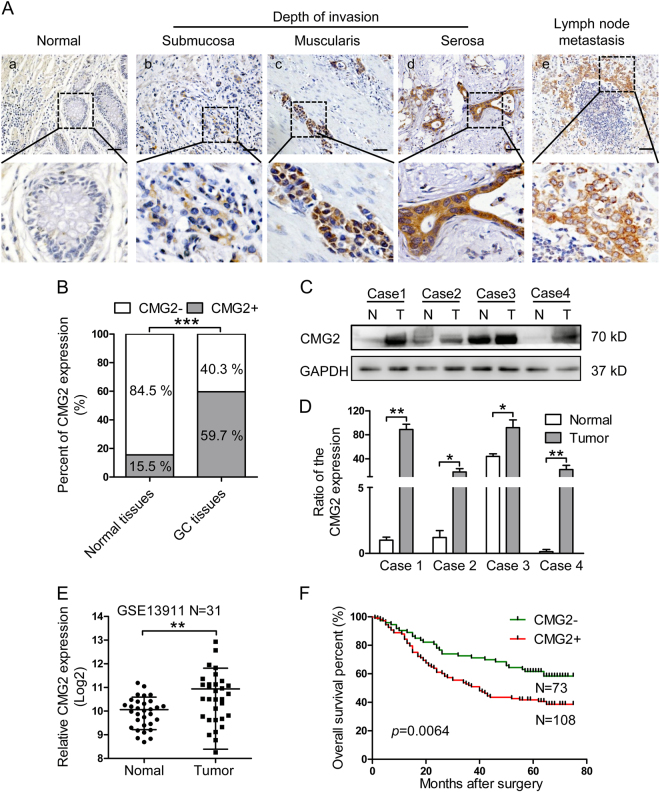
CMG2 is highly expressed in GC tissues and correlated with the outcome of patients. **a** Representative IHC images of CMG2. Scale bar = 100 μm. **a**a Absence of CMG2 expression in normal gastric mucosa. **a**b–d CMG2 staining was observed in GC tissues and the intensity was increased with the tumor invasion depth. **a**e CMG2 highly expressed in both primary tumor and the corresponding metastatic lymph node. **b** Higher percentage of CMG2+staining in GC tissues; ***P < 0.001. **c**, **d** CMG2 expression at protein and mRNA levels in four fresh surgical gastric tumor specimens (T) and paired adjacent normal tissues (N) detected by western blotting and qRT-PCR, respectively; **P < 0.01, *P < 0.05. **e** Data in the GEO database GES13911 showing higher CMG2 expression in GC tumor tissues compared to adjacent normal tissues; **P < 0.01. **f** Kaplan–Meier estimation indicating shorter overall survival time of patients with CMG2+ GC

The levels of CMG2 expression in 181 GC specimens and paired adjacent normal tissues were examined by immunohistochemistry (IHC). CMG2 staining was mainly observed in the cytomembrane and cytoplasm of GC cells (Fig. 1a). The staining of CMG2 was very low or absent in normal gastric mucosa (Fig. 1a(a)), but was high in cancer tissues as well as in metastatic lymph nodes (Fig. 1a(b–e)). As shown in Fig. 1a(b–d), the staining intensity of CMG2 was increased with the depths of tumor invasion. Among GC cancerous tissues, 108 (59.7%) were positive expression (CMG2+) and 73 (40.3%) were negative expression of CMG2 (CMG2−). In corresponding adjacent normal tissues, 153 (84.5%) showed CMG2− and only 28 (15.5%) showed CMG2+ (*p* < 0.0001, Fig. 1b). In four fresh surgical tissues of GC tumors and their corresponding adjacent normal tissues, the levels of CMG2 protein and mRNA were significantly higher in tumor tissues (T) of each case as compared to their adjacent normal tissues (N) (Fig. 1c, d). By searching a database repository of Gene Expression Omnibus (GEO), we found a transcriptional profile of 31 pair GC specimens by microarrays (GSE13911), which also showed that the mRNA expression of CMG2 was markedly higher in GC tissues than in normal tissues. This data set was analyzed and plotted in Fig. 1e (*p* = 0.0058). These date imply that higher expression of CMG2 might be associated with the progression of GC. The correlation of CMG2 expression in GC tissues with clinicopathological features was then analyzed. CMG2 expression was positively correlated with TNM stage (*p* = 0.024), T stage (*p* = 0.012), and lymph node metastasis (*p* = 0.024, Table 1). Kaplan–Meier analysis revealed that patients with CMG2+ GC had shorter life span compared to those with CMG2− GC (*p* = 0.0064, Fig. 1f). Univariate and multivariate analyses showed that CMG2 was an independent prognostic indicator for the overall survival of GC patients (*p* = 0.007 and *p* = 0.049, respectively, Table 2). These results suggest that CMG2 is involved in the GC progression and may act as a prognostic biomarker for GC.

**Table 1:** The correlation between CMG2 expression and clinical pathologic parameters of GC

Clinicopathological parameter	Total no.	CMG2		*p* value
		Negative (%)	Positive (%)	
Age, years				0.119
≤60	109	49 (45)	60 (55)	
>60	72	24 (33)	48 (67)	
Sex				0.573
Female	48	21 (44)	27 (56)	
Male	133	52 (39)	81 (61)	
T stage				0.012
T1	22	16 (73)	6 (27)	
T2	34	12 (35)	22 (65)	
T3	110	40 (36)	70 (64)	
T4	15	5 (33)	10 (67)	
Lymph node metastasis				0.024
Absent	72	38 (53)	34 (47)	
Present	109	39 (36)	70 (64)	
TNM stage				0.024
I	40	23 (58)	17 (42)	
II	59	22 (37)	37 (63)	
III	80	28 (35)	52 (65)	
IV	2	0 (0)	2 (100.0)	

*Note*: T1 indicates the localization of tumor cells in the gastric mucosa layer; T2 indicates the localization of tumor cells in gastric submucosal layer; T3 indicates the invasion of tumor cells in gastric muscular layers; and T4 indicates the invasion of tumor cells in gastric serosal layer

**Table 2:** Univariate and multivariate analyses of the contribution of CMG2 on the survival of GC patients

	Univariate analysis		Multivariate analysis	
	Hazard ratio (95% CI)	*p* value	Hazard ratio (95% CI)	*p* value
Age (>60)	1.306 (0.866–1.970)	0.202	1.147 (0.745–1.767)	0.533
Sex (male)	0.999 (0.635–1.571)	0.996	0.843 (0.524–1.356)	0.482
T stage	2.666 (1.932–3.679)	0.000	2.048 (1.349–3.109)	0.001
Lymph node metastasis	1.528 (1.290–1.811)	0.000	0.956 (0.721–1.268)	0.756
TNM	2.571 (1.916–3.450)	0.000	2.011 (1.160–3.487)	0.013
CMG2 (positive)	1.826 (1.175–2.837)	0.007	1.534 (0.984–2.391)	0.049

### CMG2^High^ cells exhibit properties of GCSLCs

**Fig. 2:**
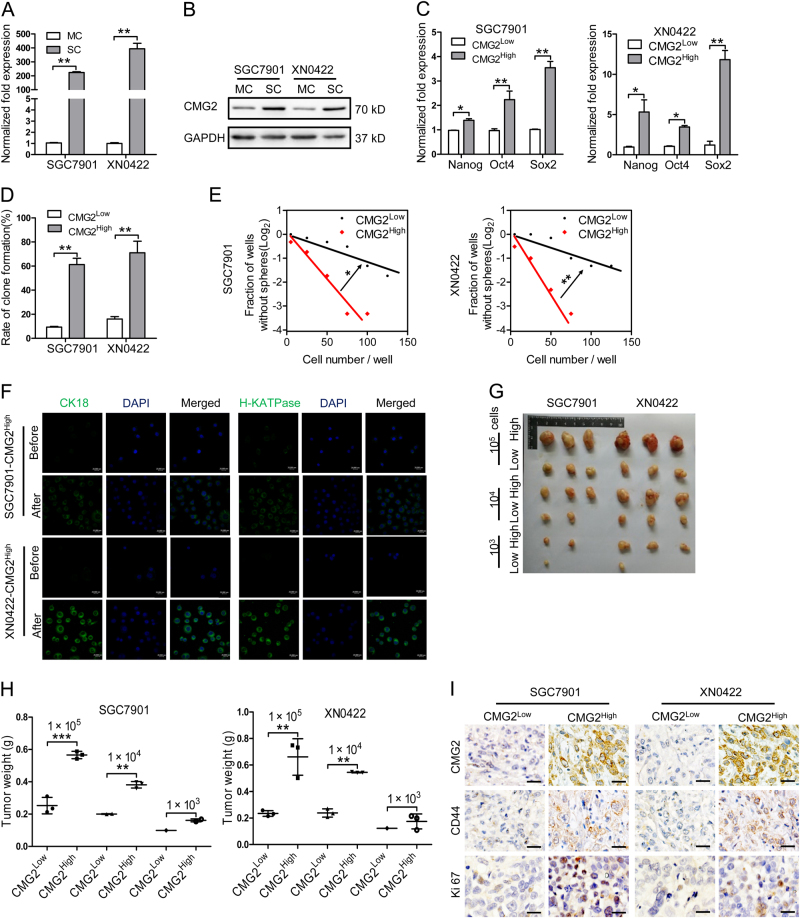
CMG2^High^ cells exhibit properties of GCSLCs. **a**, **b** qRT-RCR and western blotting showing higher CMG2 level of sphere-forming cells (SC) compared to monolayer cells (MC) in SGC7901 and XN0422 cell lines; **P < 0.01. **c** qRT-PCR showing upregulated stemness-related transcription factor genes Nanog, Oct4, and Sox2 expressed by CMG2^High^ cells compared to CMG2^Low^ cells; *P < 0.05. **P < 0.01. **d** Higher colony forming ability of CMG2^High^ cells than CMG2^Low^ cells; **P < 0.01. **e** Limiting dilution showing increased sphere formation by CMG2^High^ cells compared to CMG2^Low^ cells; *P < 0.05. **f** Supplemented with 10% FBS for 72 h, CMG2^High^ cells expressing increased differentiation markers CK18 and H-KATPase after culture with medium. **g** Formation of subcutaneous xenograft tumors by CMG2^High^ cells than CMG2^Low^ cells. **h** Increased weight of xenograft tumors formed by CMG2^High^ cells compared to CMG2^Low^ cells. **i** The xenograft tumors derived from CMG2^High^ GC cells showing higher expression of CMG2, CD44, and Ki67 compared to that derived from CMG2^Low^ cells. Scale bar = 50 μm

CMG2 expression at protein and mRNA levels in five GC cell lines were higher than in a gastric epithelia cell line, with the highest expression in SGC7901 and XN0422 (Figure S1A, B). So, SGC7901 and XN0422 cells were used for further experiments. GCSLCs are believed to be responsible for the initiation of GC [5–7]. We therefore examined the association of CMG2 with GCSLCs. We used sphere-forming capacity [20] to enrich GCSLCs from SGC7901 and XN0422 cells. Both the mRNA and protein expression levels of CMG2 were significantly higher in sphere cells (SC) than in monolayer cells (MC) (Fig. 2a, b). We then isolated CMG2-High (CMG2^High^) and CMG2-Low (CMG2^Low^) cells by fluorescence-activated cell sorting (FACS), with a percentage of CMG2^High^ cells 2.8 ± 0.4% (*n* = 3) in SGC7901 and 4 ± 0.5% (*n* = 3) in XN0422, respectively (Figure S2A). Sorted CMG2^High^ cells expressed higher levels of stemness-related transcription factors Nanog, Oct4, and Sox2 compared with CMG2^Low^ cells (Fig. 2c). Sorted CMG2^High^ cells exhibited a more potent self-renewal capacity in vitro (Fig. 2d, e and Figure S2B, S2C). Under normal culture conditions for 72 h, CMG2^High^ cells partially expressed classical differentiation markers CK18 and H-KATPase (Fig. 2f). We next compared the tumorigenicity between CMG2^High^ and CMG2^Low^ cells in nude mice. Tumors formed by CMG2^High^ cells were markedly larger than those formed by CMG2^Low^ cells, derived from both SGC7901 and XN0422 cells (Fig. 2g, h). The xenograft tumors derived from CMG2^High^ GC cells expressed higher CMG2, CD44, and Ki67 compared to that derived from CMG2^Low^ cells (Fig. 2i). The GC origin of xenograft tumors was confirmed by HE staining (Figure S3A). These results indicate that CMG2^High^ cells possess increased capabilities of self-renewal, multilineage differentiation, and tumorigenesis.

### CMG2^High^ cells are detected in CD44^High^ population both in GC cell lines and tissues

**Fig. 3:**
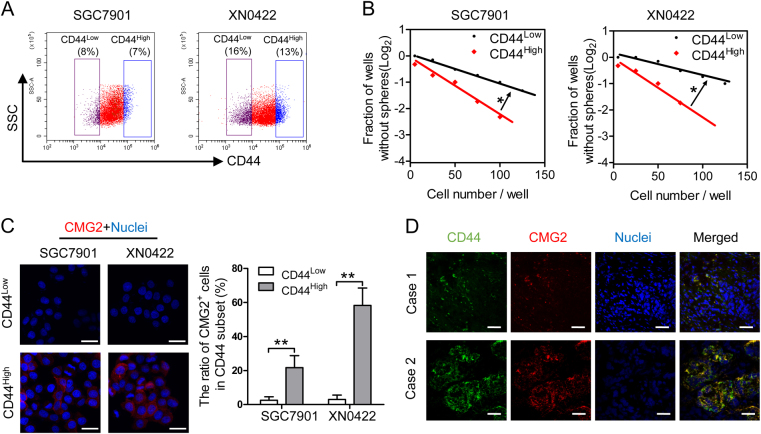
CMG2^High^ cells are detected in CD44^High^ population in GC cell lines and in GC tissues. **a** Representative flow histogram of the percentage of CD44^High^ cells in SGC7901 and XN0422 cell lines. **b** Limiting dilution showing increased capacity of sphere formation by CD44^High^ cells compared to CD44^Low^ cells; *P < 0.05. **c** Representative immunofluorescence images (left panel) and quantitative statistic results (right panel) showing more CMG2-positive cells detected in CD44^High^ subpopulation than CD44^Low^ subpopulation; Scale bar = 50 μm; **P < 0.01. **d** The co-localization of CMG2 and CD44 in the frozen sections of fresh GC tissues; Scale bar = 100 μm

To further evaluate the relationship between CMG2 and GCSLCs, we examined the expression pattern of CMG2 in relation to CD44, a well-known marker for GCSLCs [21, 22], by isolating CD44^Low^ and CD44^High^ cell populations from GC cells (Fig. 3a). Consistent with previous reports [23, 24], CD44^High^ cells exhibited increased capacity of sphere formation compared to CD44^Low^ cells (Fig. 3b). Figure 3c shows that a large number of CMG2-positive cells appeared in CD44^High^ cell population. In GC specimens, significant co-localization of CMG2 with CD44 was observed (Fig. 3d). Oncomine data analysis also showed a significant correlation between CMG2 and CD44 expression in GC (*R* = 0.934, Figure S4). Thus, CD44^High^ GCSLCs also express high level of CMG2.

### Silencing CMG2 in GC cells significantly reduces GCSLC population

**Fig. 4:**
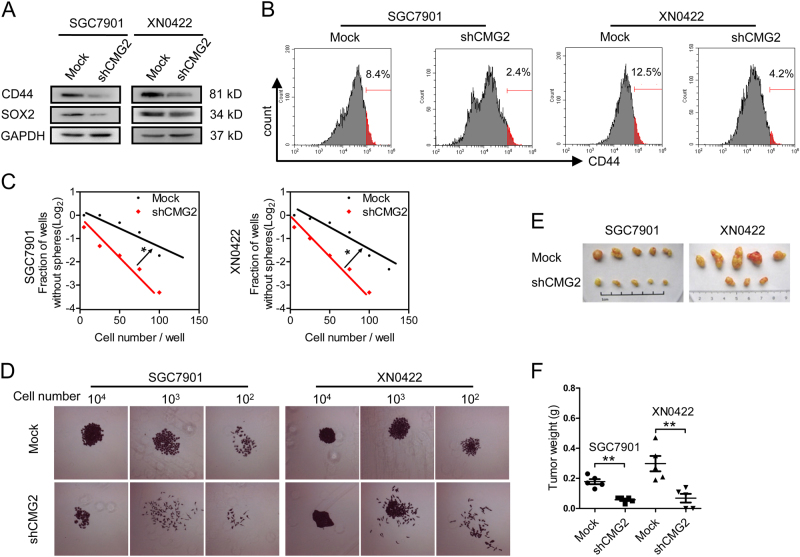
Silencing CMG2 reduces GCSLC population. **a** Decreased expression of stemness marker CD44 and stemness-related factor SOX2 in GC cells with CMG2 knockdown (shCMG2). **b** Representative flow cytometric histograms showing decreased percentage of CD44^High^ cells in shCMG2 GC cells. **c** Markedly reduced sphere formation by CMG2 knockdown GC cells; *P < 0.05. **d** Decreased colony formation by shCMG2 cells. **e** Representative images showing decreased tumor formation in nude mice by CMG2 knockdown GC cells. **f** Reduced weight of xenograft tumor formed by shCMG2 GC cells; **P < 0.01

We silenced CMG2 with shRNA in SGC7901 and XN0422 cells (Figure S5A, S5B). Loss of CMG2 significantly reduced the expression of the GCSLC marker CD44 and the stemness-related transcriptional factor SOX2 (Fig. 4a) as well as the proportion of CD44+ cells (Fig. 4b) in GC cells. CMG2 deficiency also significantly reduced the ability of sphere and colony formation by GC cells (Fig. 4c, d and Figure S6). The size of xenograft tumors derived from shCMG2 GC cells was markedly smaller than those formed by mock cells, when the same number of tumor cells was injected (Fig. 4e). H&E staining confirmed that xenograft tumors were of GC phenotypes (Figure S3B). Figure 4f shows reduced weight of xenograft tumor derived from shCMG2 GCs. These results suggest the involvement of CMG2 in maintaining GCSLCs.

### Silencing CMG2 reduces the invasiveness and metastasis of GC cells in association with reduction of epithelial–mesenchymal transition (EMT)

**Fig. 5:**
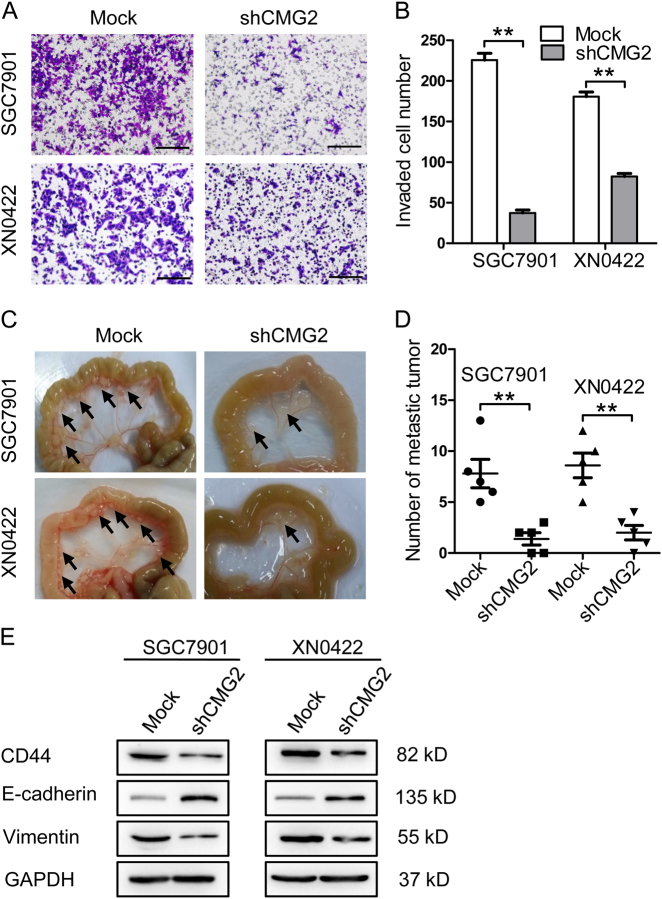
Silencing CMG2 results in reduced invasive and metastatic properties of GC cells epithelial–mesenchymal transition (EMT). **a** Representative images of transwell invasion test showing decreased invasion capability of GC cells with knockdown of CMG2. **b** Quantification of the transwell invasion assay results; Scale bar = 100 μm; **P < 0.01. **c** Representative images of intraperitoneal metastasis tests showing reduced number of metastatic foci formed by shCMG2 cells. **d** Quantification of the intraperitoneal metastasis results; **P < 0.01. **e** Western blot showing upregulation of E-cadherin and downregulation of CD44 and vimentin in GC cells with knockdown of CMG2

We further evaluated the effect of silencing CMG2 on the invasive and metastatic capacities of GC cells. Knockdown of CMG2 significantly diminished the invasiveness of GC cells (Fig. 5a, b), while overexpression of CMG2 (Figure S5C, D) significantly elevated the invasive capability of GC cells (Figure S7). In an intraperitoneal metastasis model that allows for visualization of metastatic foci in the abdominal cavity and mesentery of mice, the occurrence of metastatic foci was significantly lower in nude mice implanted with shCMG2 cells as compared with mock cells (Fig. 5c, d). In evaluating the effect of CMG2 depletion on EMT-associated markers E-cadherin and vimentin, we found that CMG2-depleted GC cells showed upregulation of E-cadherin but downregulation of vimentin as well as reduction of CD44 (Fig. 5e). Thus, CMG2 enhances the invasiveness and metastasis of GCSLCs by inducing EMT.

### CMG2 interacts with LRP6 to activate a Wnt/β-catenin pathway in GC cells

**Fig. 6:**
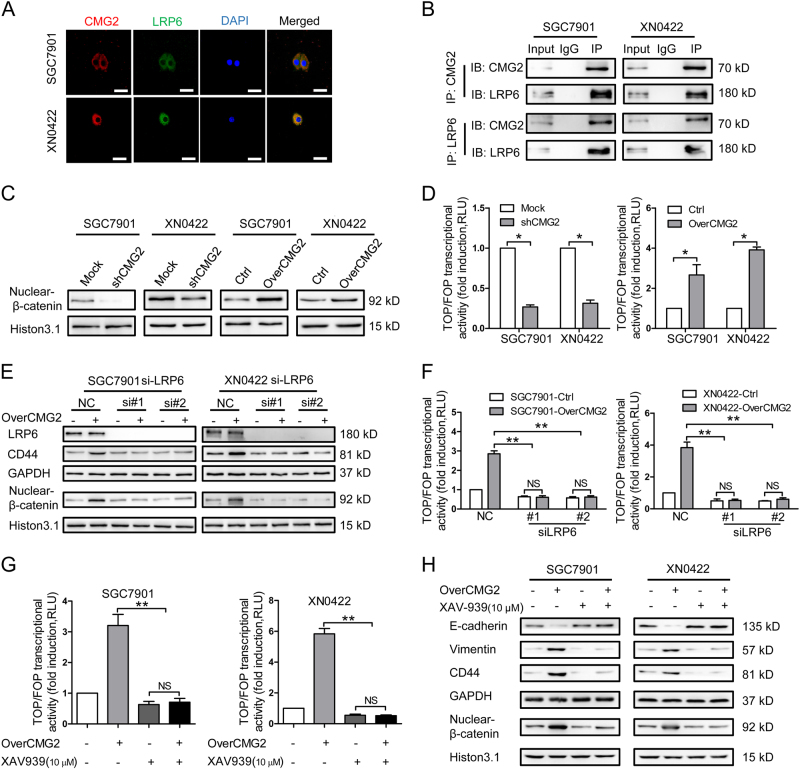
CMG2 interacts with LRP6 to activate β-catenin pathway in GC cells. **a** Representative immunofluorescence images showing co-localization of CMG2 and LRP6 in GC cells; Scale bar = 50 μm. **b** Co-IP confirmation of physical interaction between CMG2 and LRP6. **c** Western blotting showing reduced level of nuclear β-catenin in shCMG2 GC cells. **d** TOP/FOP flash showing decreased transcriptional activity of TCF4 in GC cells with silenced CMG2 (left panel) but increased transcriptional activity of TCF4 in GC cells with overexpressed CMG2 (left panel); *P < 0.05. **e** Western blot showing attenuated nuclear β-catenin level siLRP6 GC cells. **f** TOP/FOP flash showing decreased transcriptional activity of TCF4 in siLRP6 GC cells; **P < 0.01. **g** Treatment with XAV-939, a specific inhibitor of β-catenin, abolishing CMG2 overexpression-induced TCF4 transcriptional activity; **P < 0.01. **h** Treatment with XAV-939 decreasing nuclear accumulation of β-catenin, downregulating CD44 and vimentin, and upregulating E-cadherin in overCMG2 GC cells

To investigate the mechanism by which CMG2 regulates the stemness of GCSLCs, we searched String database for putative interacting proteins of CMG2, in which a total of 10 proteins were predicted (Figure S8). Among these proteins, low-density lipoprotein receptor 6 (LRP6) is a key component of the Wnt/β-catenin signaling pathway. We therefore tested whether CMG2 may maintain the stemness of GSCLCs through CMG2–LRP6 interaction to activate Wnt/β-catenin signaling. As shown in Fig. 6a, CMG2 and LRP6 were co-localized in GC cells. Co-IP demonstrated a physical interaction between CMG2 and LRP6 (Fig. 6b, upper panel), which was also confirmed by Co-IP using an anti-LRP6 antibody (Fig. 6b, lower panel). To further investigate the capacity of CMG2 to activate Wnt/β-catenin pathway in conjugation with LRP6, we observed the nuclear translocation of β-catenin. Silencing CMG2 significantly reduced the level of β-catenin in the nucleus of GC cells (Fig. 6c, left panel), while overexpressing CMG2 markedly enhanced nuclear β-catenin in GC cells (Fig. 6c, right panel). TOP flash and FOP flash were then used to evaluate β-catenin-dependent signaling events that promote the expression of TCF in GC cells [25, 26]. Depletion of CMG2 reduced β-catenin signaling (Fig. 6d, left panel), while overexpression of CMG2 significantly upregulated β-catenin signaling in GC overexpressing cells (Fig. 6d, right panel), suggesting changes in CMG2 levels in GC cells substantially affected β-catenin signaling. We then depleted LRP6 both in CMG2 overexpression and control GC cells to observe the change of CD44 expression and nuclear β-catenin levels. Compared with control cells (NC), the levels of CD44 and nuclear β-catenin in siLRP6 cells were significantly reduced. Moreover, LRP6 depletion nearly abolished the effect of CMG2 overexpression on upregulated levels of CD44 and nuclear β-catenin (Fig. 6e). TOP/FOP assay showed the requirement of LRP6 for CMG2-mediated β-catenin signaling (Fig. 6f). Furthermore, changes in CMG2 and LRP6 levels resulted in consistent alteration of CD44 and nuclear β-catenin levels. Treatment with XAV-939 (10 μM), a specific inhibitor of β-catenin [27], resulted in reduced nuclear accumulation of β-catenin, decreased TOP/FOP transcriptional activity, downregulated CD44 and vimentin, and upregulated E-cadherin in overCMG2 GC cells (Fig. 6g, h). These results indicate that CMG2 regulates Wnt/β-catenin pathway through LRP6 to maintain the stemness of GCSLCs.

## Discussion

In this study, we found that CMG2 is highly expressed in GC tissues and the expression levels are correlated with the invasion depth and lymph node metastasis, as well as poor survival of the patients. This is the first demonstration of the link between CMG2 and the clinicopathological features of GC and the outcome of patients.

CMG2 is originally identified as a gene upregulated in endothelial cells during capillary morphogenesis [8]. It has been implicated in tumor-related angiogenesis [28] and may be a target for anti-angiogenesis therapy for cancer [15, 29, 30]. However, the role of CMG2 in cancer has not been extensively investigated. Zou et al. [19] reported that cholesterol depletion promoted CMG2 expression, which was responsible for ERK phosphorylation and activation of MMP-2 in neuroglioma cells, implying involvement of CMG2 in the invasiveness of tumor cells. Ye et al. [17] found that CMG2 enhanced the adherence, inhibited the invasiveness, but did not affect the growth of prostate cancer cells. Ye et al. [19] also found that CMG2 inhibited the growth of breast cancer cells and the expression level in breast cancer tissues was with earlier disease stages and favorable overall patient survival. The reason for these contradictory results is not clear but may be attributed to the difference in tumor types.

GCSLCs possess the capacities of self-renewal, differentiation, and generating tumors, and are responsible for progression of GC, including invasion, metastasis, resistance to chemoradiotherapy, and recurrence [31, 32]. The key molecules and signaling pathways underlying GCSLC persistence are not clear. Recently, GCSLC surface markers have been identified including CD44 [23, 33], CD133 [34], CD90 [35], and LGR5 [36]. CD44 is considered as a more reliable marker for GCSLCs as a transmembrane glycoprotein. Takaishi et al. [23] and others [24, 37, 38] showed that CD44^High^ cells constitute an important GCSLC population. We found that CMG2 is preferentially expressed in CD44^High^ stem-like cell population. When CMG2 was depleted from GC cells, the size of CD44^High^ population was markedly reduced. CMG2-depleted GC cells showed diminished stemness and tumorigenicity. Thus, CMG2 appears to be important in maintaining GCSLC population.

Several signaling pathways have been implicated in the maintenance of human GCSLCs, including Wnt/β-catenin, Hedgehog, and Notch pathways [39–45]. Wei et al. [46] reported that LRP6 interacts with CMG2 to mediate the entry of PA/receptor complexes into the cytoplasm and the lethality of anthrax toxin. LRP6 is initially identified as a member of low-density lipoprotein receptor family localized on the cell surface and mediates endocytosis of lipids [47, 48]. However, it has also been demonstrated that LRP6, as an essential Wnt co-receptor, mediates the transduction of signals from secreted Wnt proteins to β-catenin [49, 50]. These observations suggest that CMG2 may maintain on GCSLCs through activating Wnt/β-catenin signaling pathway. Our study confirms the physical interaction between CMG2 and LRP6, and thus illustrates a novel regulative pathway in GCSLCs.

EMT is a highly conserved and critical for embryogenesis, wound healing, fibrosis, stem cell biology, and tumor progression [51, 52]. During EMT, epithelial cells lose cell-to-cell contact and polarity to undergo cytoskeletal remodeling, acquiring increased motility and invasiveness [53, 54]. In GC, EMT is associated with increased capability of tumor cell invasion and metastasis [55, 56]. Recently, the connection between EMT and the acquisition of stem-like properties were described in multiple tumor types [57–60]. Bessède et al. [61] found that Helicobacter pylori generates cells with GCSLC properties via EMT in GC. Ryu et al. [62] demonstrated that CD44 expression was significantly associated with the expression of EMT-related proteins. We also showed that GCSLCs undergoing EMT are more invasive and metastatic than other GC cells [20]. Our current study suggests that EMT is an important mechanism for CMG2 to promote GCSLC invasion and metastasis.

Since CMG2 is the most important receptor for anthrax toxin and plays an important role in angiogenesis, targeting CMG2 represents a novel therapeutic strategy for anthrax infection and cancer progression. However, since CMG2 is also expressed in normal tissues and blood vessels, targeting the pathway of CMG2 and LRP6 interaction may be more specific for eliminating GCSLCs.

It is noteworthy that CMG2 contains four isoforms [63], including CMG2^489^, CMG2^488^, CMG2^386^, and CMG2^322^. Our work revealed that only CMG2^489^ and CMG2^488^ isoforms were elevated, but not CMG2^386^ and CMG2^322^ in GC tissues and cell lines (Figure S1A, B, C).

In summary, this study demonstrated that CMG2 promotes GC progression by maintaining GCSLCs and can serve as a new prognostic indicator and a target for human GC therapy. CMG2 maintains GCSLCs thought a novel regulative process on Wnt/β-catenin pathway.

## Materials and methods

### Patients

Surgical specimens of cancerous tissues and paired adjacent normal tissues were collected from 181 patients with GC between 2006 and 2007 (Southwest Hospital, Chongqing, China). No preoperative history of radiotherapy or chemotherapy was reported in any of the patients. Written consents for the biological studies were obtained from the patients or their guardians. According to the WHO standard, each specimen was histologically examined and graded by two experienced pathologists. This study was approved by the Ethics Committee of the Southwest Hospital.

### IHC

IHC staining was performed according to the manufacturer’s instructions of the Dako REAL EnVision Detection System (Dako). The following are the primary antibodies used in IHC: CMG2 antibody that recognizes all isoforms of CMG2 (Cat. no. 16723-1-AP, Proteintech) or CD44 antibody that recognizes all isoforms of CD44 (Cat. no. BBA10, R&D) or Ki67 antibody (Cat. no. F7268, Dako). Two pathologists in a blinded manner independently evaluated all slides. Tumor and normal tissues were categorized as CMG2-positive and -negative according to whether cancer cells with CMG2 staining was ≥5%.

### Cell lines and cell culture

Primary GC cell line XN0422 was established in our laboratory [64]. Human GC cell lines AGS, HGC27, MGC803, and SGC7901 and gastric epithelial cell GES-1 were purchased from Chinese Academy of Sciences Cell Cultures Library (Shanghai, China). AGS was cultured in F12K medium (containing 10% FBS, Gibco) at 37 °C in 5% CO_2_ and 100% humidity. Other GC cells and GES-1 were cultured in RPMI1640 medium (containing 10% FBS, Gibco) at 37 °C in 5% CO_2_ and 100% humidity.

### FACS

Single GC cell suspension was prepared by trypsinization of cultured adherent cells and stained with APC-conjugated anti-human CMG2 antibody that recognizes all isoforms of CMG2 (Cat. no. FAB2940A, R&D) or PE-conjugated anti-human CD44 antibody that recognizes all isoforms of CD44 (Cat. no. 550989, BD) for 30 min at room temperature followed by FACS analysis (BD FACS Aria II). Forward side scatter and pulse-width gating were used for excluding the dead cells, debris, and aggregates. Isotypes-matched primary antibodies were used as controls.

### Colony formation assay

For analysis of colony formation, 200 viable GC cells were equably seeded in each well of 6-well plates and cultured in the RPMI1640 medium (containing 10% FBS). After incubating for 14 days and staining with crystal violet, the colonies which contained more than 50 cells were counted.

### Limiting dilution assay

Serial twofold dilutions of GC cells (from 100 to 0) were seeded into ultra-low adhesion 96-well plates (10 wells per dilution, Costar, USA) and cultured in the stem cell medium (serum-free DMEM/F12 medium with EGF (20 ng/ml, Sigma, USA) and B27 (1×, Invitrogen, USA)) at 37 °C in 5% CO_2_ and 100% humidity. Fresh medium (20 μl) was added to each well every 3 days. After incubation for 10 days, wells without spheres (log_2_, *y*-axis) were counted and plotted against the number of cells plated per well (*x*-axis) to calculate the sphere formation efficiency.

### Quantitative real-time PCR (qRT-PCR)

Total RNA of GC cells was extracted with RNAiso reagent (Takara, Japan), and reverse-transcribed with PrimeScript™ RT Master Mix (Takara, Japan) according to the manufacturer’s instructions. Then, a SYBR® Fast qPCR Mix (Takara, Japan) in a Bio-Rad CFX96 Real-Time PCR Detection System (Bio-rad) was used for qRT-PCR. The expression of genes was determined using a 2^−△△CT^ method. qRT-PCR was performed in triplicate and the results were normalized against GAPDH. All primer sequences for qRT-PCR were listed in Table S3.

### In vitro cell invasion assay

Matrigel transwell analyses were performed as previously described [65]. Briefly, we vested transwell chambers (8 µm pore size, Millipore) with mixed matrigel (matrigel and RPMI1640,1:3, v/v). GC cells were implanted at 5 × 10^4^ cells/well and incubated for 1 day. The invasive cells were stained and then counted from five random visual areas at 100-fold magnification under a microscope.

### Multilineage differentiation assay

To determine cell differentiation of GCSLCs, isolated GC cells were cultured in RPMI1640 medium (containing 10% FBS) for 72 h. H-K-ATPase, a parietal cell marker and CK18, an epithelial cell marker, were then examined by confocal laser scanning microscopy.

### Subcutaneous tumorigenicity and intraperitoneal metastasis assays

For subcutaneous tumorigenicity assay, the 6-week-old female nude mice were grouped randomly and double blindly (*n* ≥ 3 in each group), then differently treated GC cells were injected subcutaneously into axilla of the mice. At the end of 5 weeks after the injection, the mice were killed. Xenograft tumors were removed and weighted.

For intraperitoneal metastasis assay, 1 × 10^5^ GC cells were injected intraperitoneally into nude mice (*n* = 5 in each group). At the end of 4 weeks, the mice were killed and the metastatic foci were counted. All animal procedures in this study were approved by the Third Military Medical University (Army Medical University) Animal Committee.

### Confocal laser scanning microscopy

GC cells or frozen GC tissue sections were fixed for 15 min in 4% paraformaldehyde. After washing with PBS, the sections were blocked with a protein-blocking solution. A rabbit monoclonal antibody against LRP6 (Cat. no. ab134146, Abcam) or a goat polyclonal antibody that recognizes all isoforms of CMG2 (Cat. no. SAB2501374, Sigma-Aldrich) or rabbit monoclonal antibody against CD44 (Cat. no. BBA10, R&D) or CK18 (Cat. no. ab82254, Abcam) or H-KATPase (Cat. no. ab2866, Abcam) was added onto the slides. After incubation overnight at 4 °C, the sections were incubated with Alexa Fluor® 488 goat anti-rabbit IgG (H+L) (Invitrogen, Carlsbad, CA) and/or Alexa Fluor® 647 donkey anti-goat IgG (H+L) at 37 °C for 30 min. Cell nuclei were then stained with Hoechst 33342. All samples were then analyzed by a confocal laser scanning microscopy.

### Western blot

Immunoblot analyses were performed as previously described [66]. The primary antibodies used in this study were as follows: anti-CMG2 (Cat. no. 16723-1-AP, Proteintech) or anti-GAPDH (Cat. no. 5174, CST) or anti-SOX2 (Cat. no. 3579, CST) or anti-CD44 (Cat. no. BBA10, R&D) or anti-E-cadherin (Cat. no. 3195, CST) or anti-vimentin (Cat. no. 5741, CST) or anti-β-catenin (Cat. no. 8480, CST) or anti-histone 3.1 (Cat. no. 4499, CST).

### Co-immunoprecipitation

Co-IP was performed using a Thermo Scientific Pierce Co-IP kit (Thermo Scientific, Watertown, MA, USA) following the manufacturer’s instruction. Briefly, 10 μg anti-CMG2 antibody (Cat. no. 16723-1-AP, Proteintech) or anti-LRP6 antibody (Cat. no. ab75358, Abcam) and matched IgG were immobilized for 2 h using AminoLink Plus coupling resin, respectively. Then, the resin was washed with wash solution and incubated with 250 μg GC cell lysate at 4 °C overnight. After incubation, the resin was washed again and protein was eluted using elution buffer.

### CMG2 knockdown and overexpression in GC cells

The sequences containing an effective shRNA-targeting CMG2 and a scramble (mock) were listed in Table S1. Lentivirus particles containing shCMG2 and mock shRNA were prepared by Sbo-Bio Co. Ltd (Shanghai, China) and used to infect SGC7901 and XN0422 GC cells. Then, stably transfected GC cells were selected using FACS. For overexpressing CMG2 in GC cells, lentiviral particles containing human full-length CMG2 was prepared and used to infect SGC7901 and XN0422 cells. Stable CMG2 overexpressing (overCMG2) and control cells were selected using 3 μg/ml puromycin.

### RNA interference

siRNAs targeting LRP6 and a scramble sequence were listed in Table S2. To silence human LRP6, GC cells were transfected for 48 h with 25 pmol siRNA or control RNA/well in 6-well plates using Lipofectamine® RNAiMAX Reagent (Invitrogen).

### Luciferase reporter assay

Luciferase reporter assay was performed as previously described [67]. Briefly, total DNA (TOP flash-gene or FOP-flash reporter DNA and Renilla reporter) was transfected in GC cells by using Lipofectamine 2000 (Invitrogen). Then, a Dual-Luciferase Reporter Assay System (Promega) was used for measuring the luciferase activity of GC cells treated with or without XAV-939 (10 μM) for 48 h. After normalization against Renilla, the ratio of TOP/FOP flash was conducted as the results.

### Statistical analysis

The results from representative experiments are presented. Student’s *t* test using SPSS 20.0 software (SPSS Inc., Chicago, IL, USA) and GraphPad Prism 5 (GraphPad, La Jolla, CA, USA) was used for statistical analysis of mean ± SD. The relationship between GC clinicopathological features and CMG2-positive rate was evaluated by Chi-square analysis. The OS of GC patients was estimated by using Kaplan–Meier method. Cox’s proportional hazard regression model was established for univariate and multivariate analyses of the combined contribution of CMG2 and clinicopathological features to the OS of patients. All experiments were conducted at least three times. *P* < 0.05 was considered as statistically significant.

## Electronic supplementary material

Supplementary Figures and Tables(DOC 16727 kb)
