# Supplementary material for: Capillary morphogenesis gene 2 maintains gastric cancer stem-like cell phenotype by activating a Wnt/β-catenin pathway
Source: Oncogene. 2018 Apr 17;37(29):3953–66. doi: 10.1038/s41388-018-0226-z (PMC6053357; doi:10.1038/s41388-018-0226-z)
Supplement: Supplementary file 1 — Supplementary Figures and Tables(DOC 16727 kb) [file 41388_2018_226_MOESM1_ESM.doc]

Supplementary tables

Table S1. Sequences of CMG2 sh and mock RNAs

| Marker | Name | Target Sequence | GC% |
| --- | --- | --- | --- |
| pLVT713 | shCMG2 | GCTCAGTCATGTACTGAAA | 42.1 |
| pLVT4 | Mock | TTCTCCGAACGTGTCACGT | 52.6 |

Table S2. Sequences of LRP6 si and NC RNAs

| Name | Target Sequence |
| --- | --- |
| siLRP6#1 | GCAGAUAUCAGACGAAUUUUU |
| siLRP6#2 | CAGAUGAACUGGAUUGUUAUU |
| NC | GCUCAACCGUGAAGUUAUAUU |

Table S3. The primer sequences for qRT-PCR in the experiments

| Genes | Primers sequences (5’- 3’) | |
| --- | --- | --- |
|  | Forward Reverse | |
| All CMG2s | GCTAGTGTTTATTGTGTTGGTGTCCTTGA | AGAATTAATTATTCCTTTAAGAGCCTGAAA |
| CMG2^489^ | TTTGTATATGGGAATGTATTGAGAAA | CCTTCCTCAAGTGCAATAGGGCTTTAAA |
| CMG2^488^ | CTGAGGAAGGTGCAAGGCTAGAGAAAGC | CTTTCTCAATACATTCCCATA |
| CMG2^386^ | GGCTCTTAAAGGAATAATTAATTCT | TTACTGAGATGGAACTCGGGAGAAGTTTATGCACCGGC |
| CMG2^322^ | CTGAAATCCTAGAATTGCAGCCCTCAAGTG | TCAGGAGGCTGAAGTAGGAGGATCA |
| NANOG | TTTGTGGGCCTGAAGAAAACT | AGGGCTGTCCTGAATAAGCAG |
| OCT4 | GCAGCGACTATGCACAACGA | CCAGAGTGGTGACGGAGACA |
| SOX2 | CATCACCCACAGCAAATGACA | GCTCCTACCGTACCACTAGA  ACTT |
| GAPDH | AGCCACATCGCTCAGACA | GCCCAATACGACCAAATCC |

Supplementary Figures


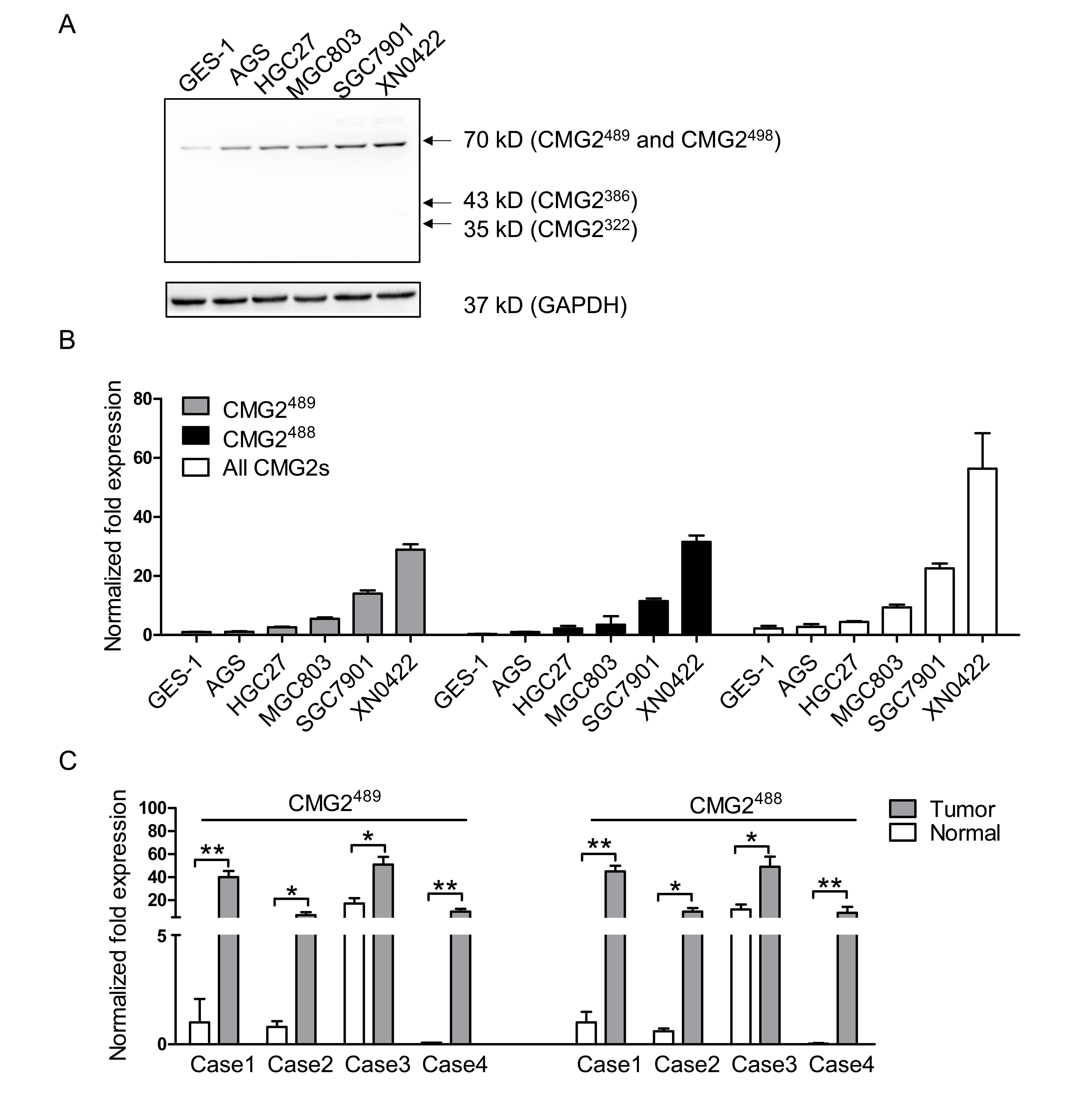


**Figure S1.** The expression of CMG2 and their isoforms in human GC cell lines and tissues. (A) Imaging of Western blotting showing the expression of CMG2 and their isoforms in 5 human GC cell lines and a gastric epithelia cell line. (B) The expression of total CMG2 and its isoforms at mRNA level measured by qRT-PCR (normalized against GAPDH) in 5 human GC cell lines and a gastric epithelia cell line. (C) The expression of CMG2 isoforms at mRNA level measured by qRT-PCR (normalized against GAPDH) in GC and paired adjacent normal tissues.


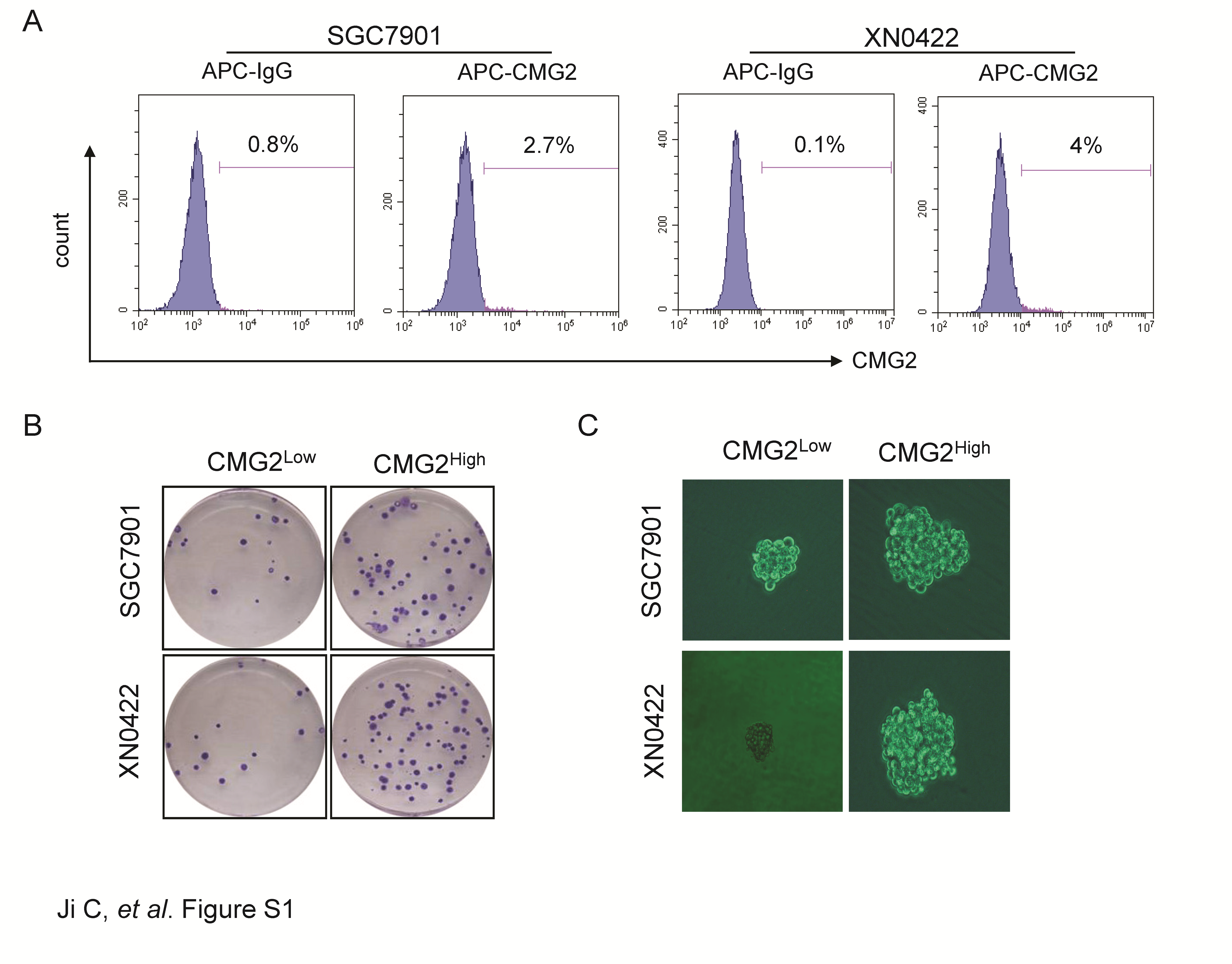


**Figure S2.** Representative images of CMG2^High^ GC cell sorting and colony and sphere formation by CMG2^High^ and CMG2^Low^ GC cells.

(A) Representative flow histogram showing the percentage of CMG2^High^ population in GC cells. (B and C) Representative images of colony and sphere formation by CMG2^High^ and CMG2^Low^ GC cells.


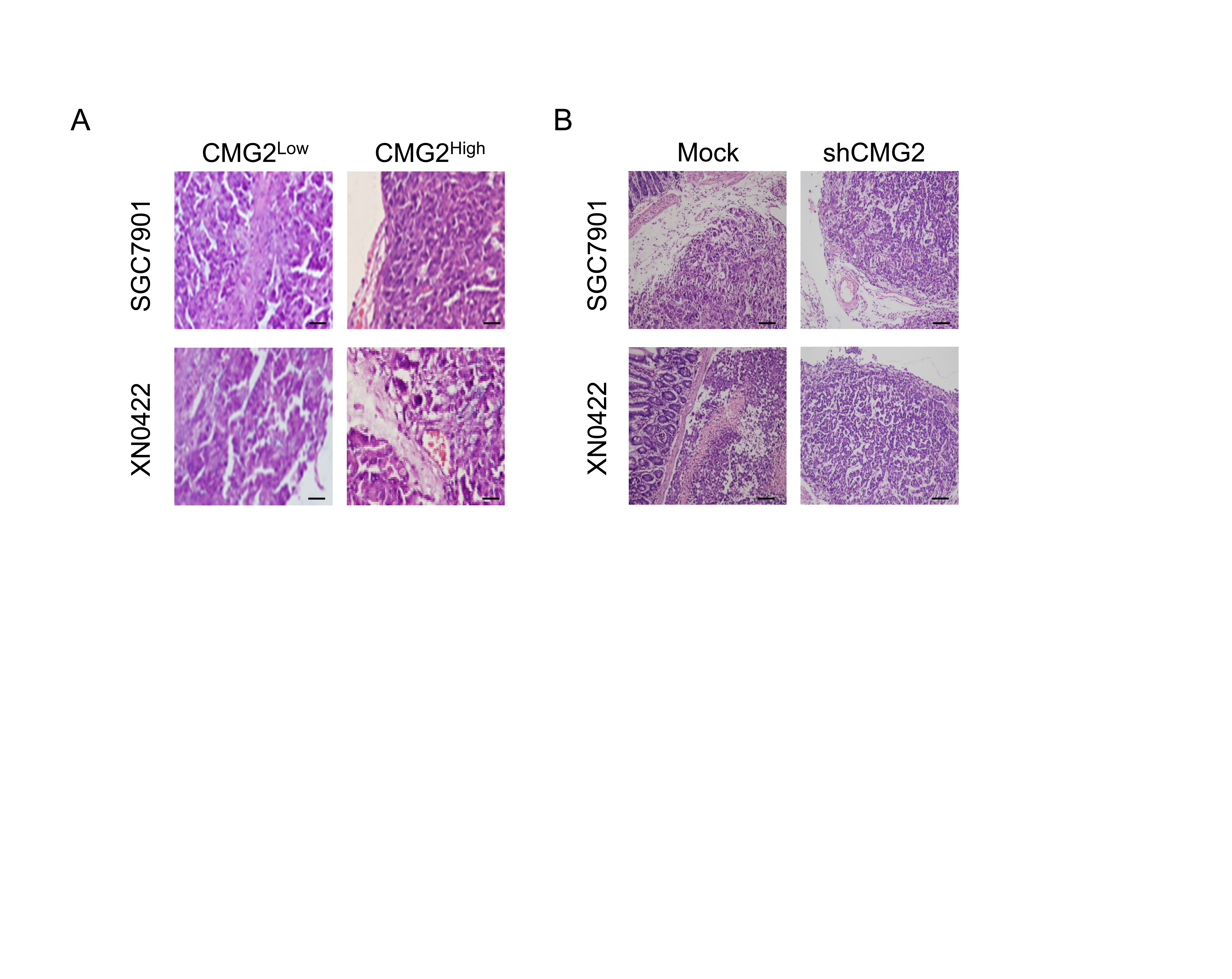


**Figure S3.** The GC nature of xenograft tumors confirmed by H&E staining.

(A) H&E staining showing the GC nature of xenograft tumors derived from CMG2^High^ and CMG2^Low^ GC cells. Scale bar = 50 μm (B) H&E staining showing the GC nature of xenograft tumors derived from mock and shCMG2 GC cells. Scale bar = 100 μm


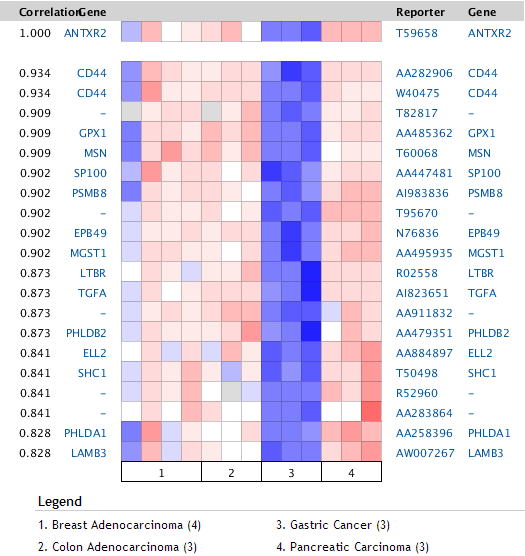


**Figure S4.** Oncomine data analysis shows significant correlation between CMG2 and CD44 expression.


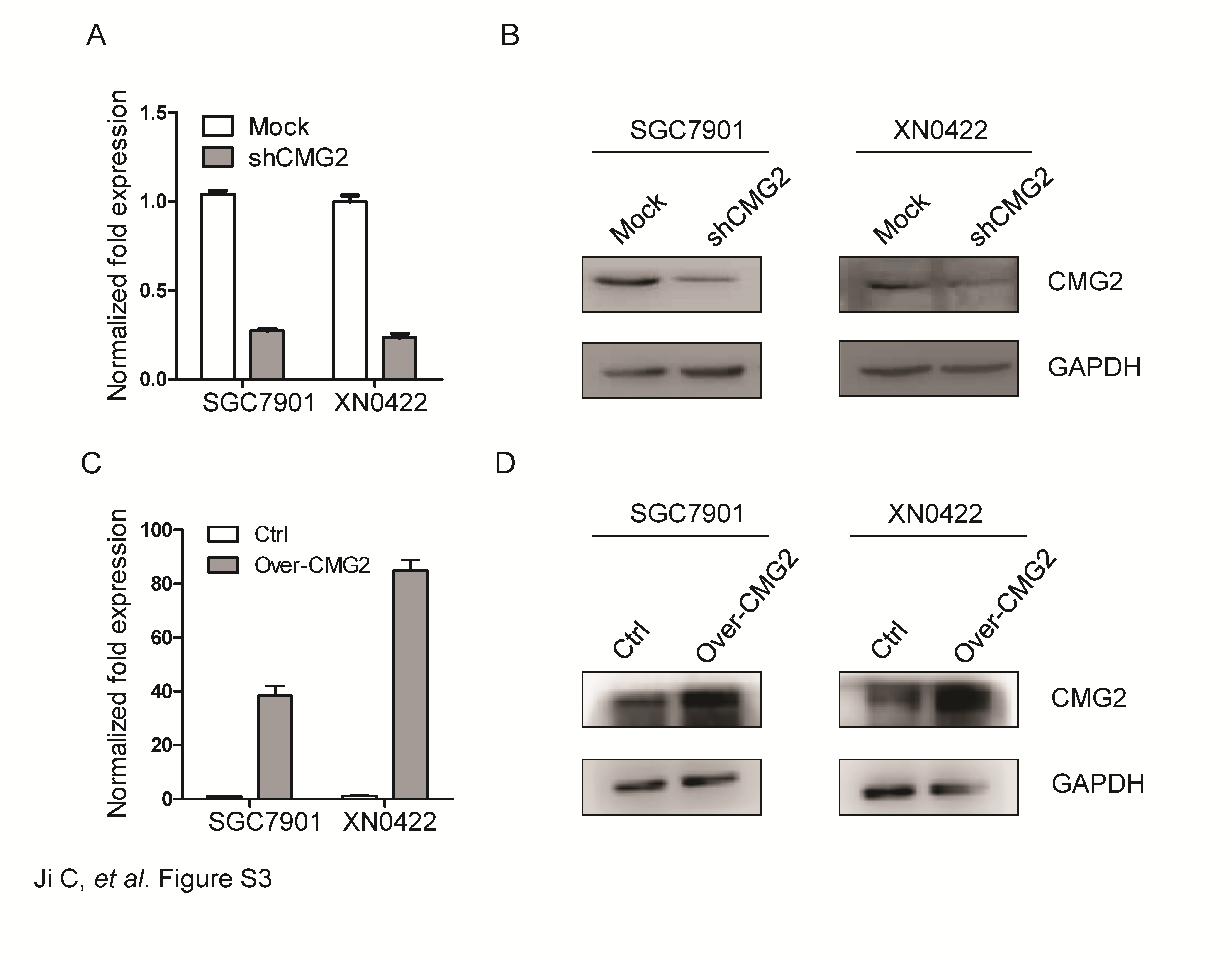


**Figure S5.** The efficiency of CMG2 knockdown and over-expression in GC cells*.*

(A and B) The efficiency of CMG2 knockdown in SGC7901 and XN0422 cells at mRNA level (A) and protein levels (B). (C and D) The efficiency of CMG2 overexpression in SGC7901 and XN0422 cells at mRNA level (C) and protein levels (D).


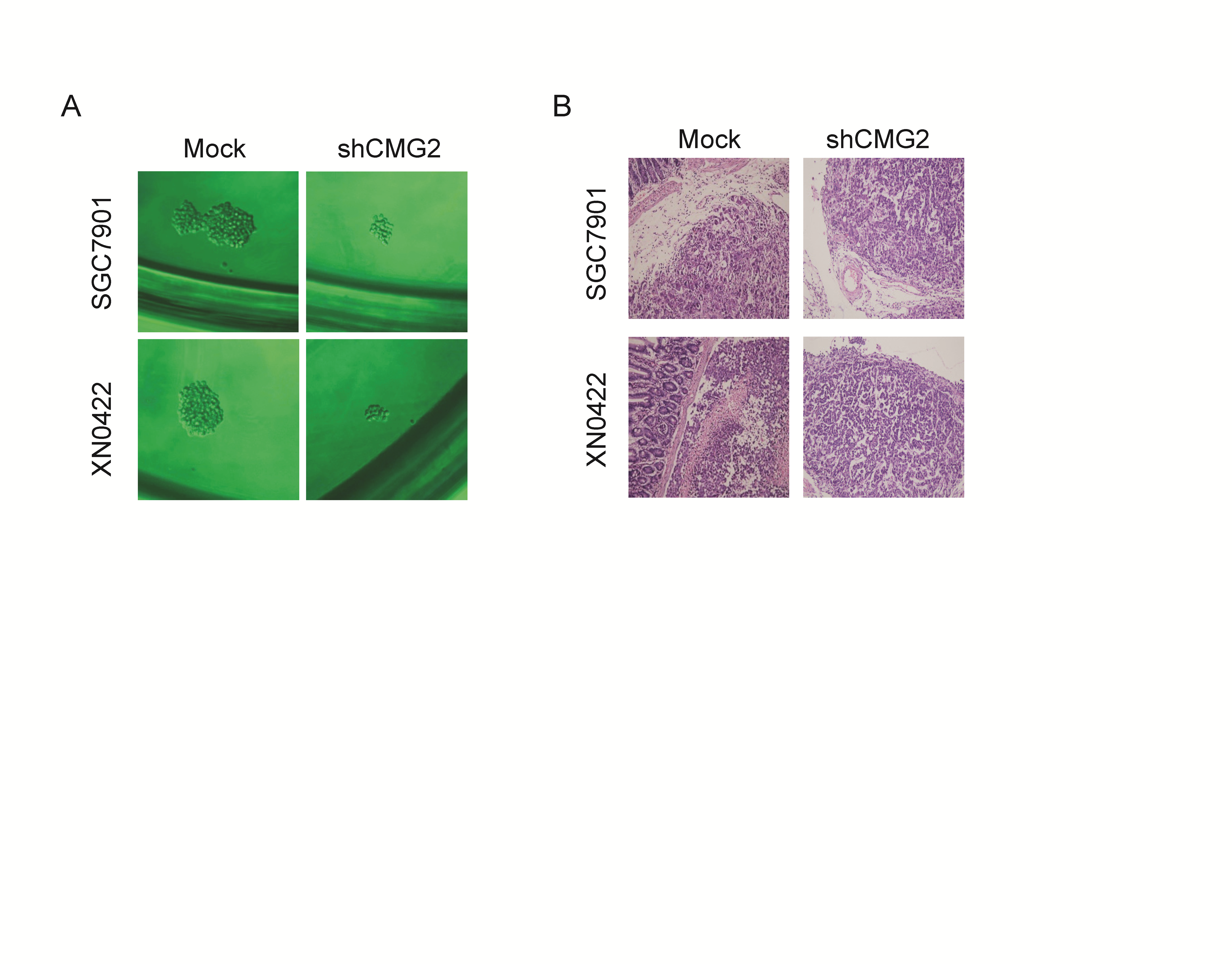


**Figure S6.** Representative images of sphere formation by XN0422 and SGC7901 cells with or without CMG2 knockdown.

**
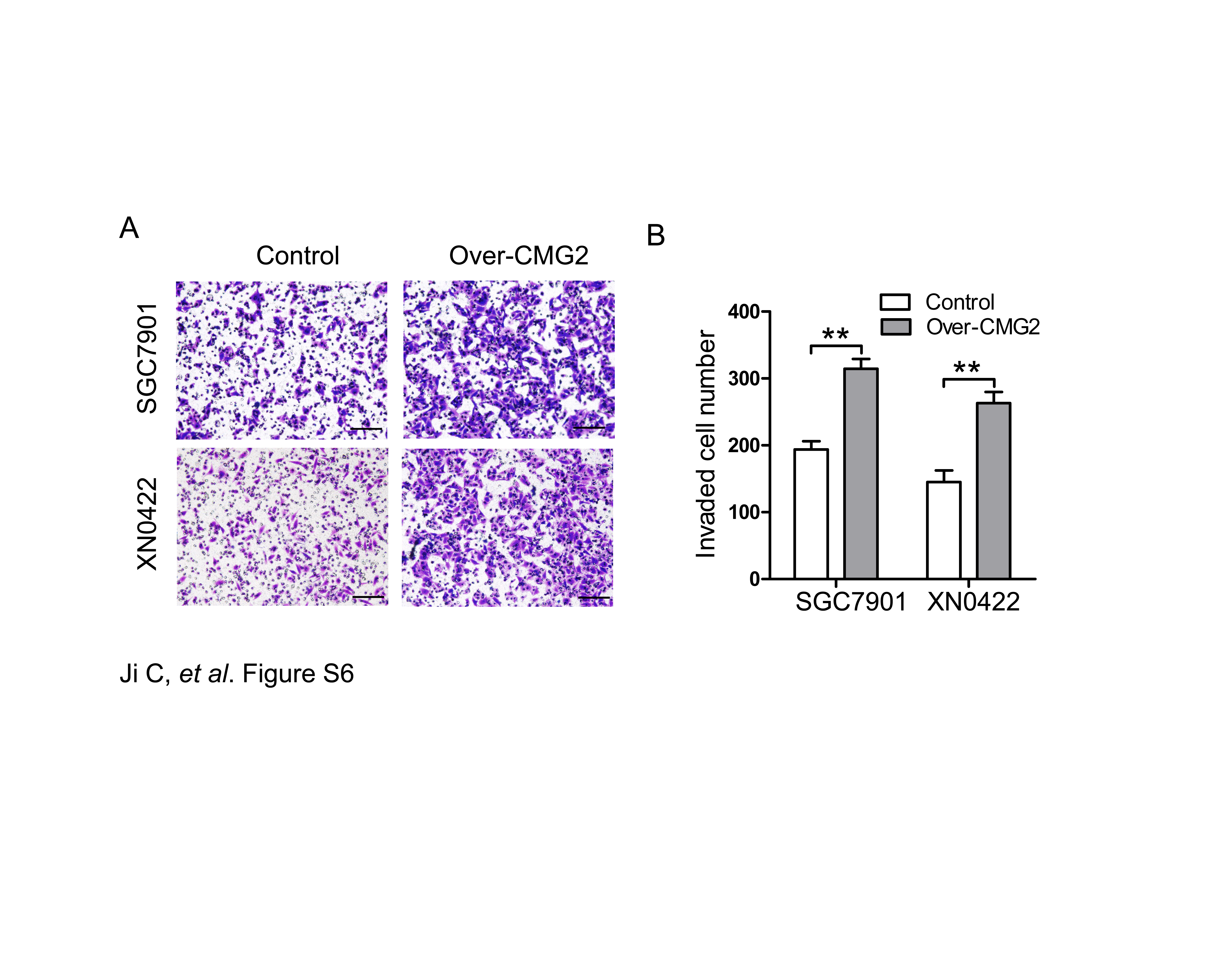
**

**Figure S7.** Overexpressing CMG2 enhances the invasiveness of GC cells.

(A) Representative images of transwell invasion test showing increased invasion capability of GC cells by overexpression of CMG2; Scale bar=100 μm. (B) Quantification of the transwell invasion assay results; **, *P* < 0.01.


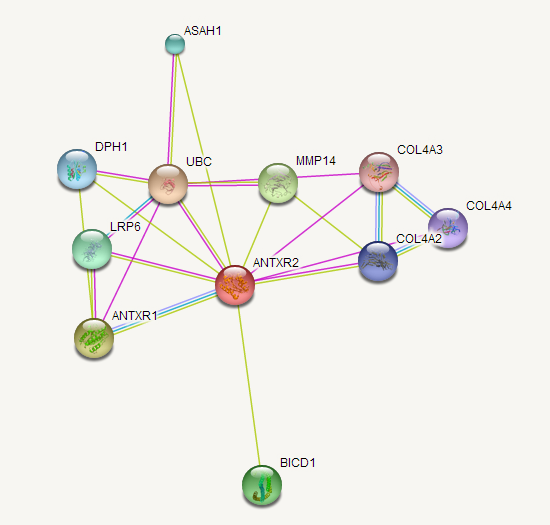


**Figure S8.** Putative interacting proteins with CMG2 described in String database.
